# Tailings after Iron Extraction in Bayer Red Mud by Biomass Reduction: Pozzolanic Activity and Hydration Characteristics

**DOI:** 10.3390/ma14143955

**Published:** 2021-07-15

**Authors:** Yaguang Wang, Xiaoming Liu, Yong Li, Dongsheng Li, Wei Zhang, Yang Xue

**Affiliations:** 1 State Key Laboratory of Advanced Metallurgy, University of Science and Technology Beijing, Beijing 100083, China; wangyg@xs.ustb.edu.cn; 2 School of Metallurgical and Ecological Engineering, University of Science and Technology Beijing, Beijing 100083, China; xkliyong@bgrimm.com (Y.L.); b20160089@xs.ustb.edu.cn (D.L.); b20190124@xs.ustb.edu.cn (W.Z.); cdxueyang@163.com (Y.X.)

**Keywords:** bayer red mud, pozzolanic activity, cementitious materials, hydration characteristics, solid waste, polymerization degree

## Abstract

Bayer red mud (BRM) is a kind of solid waste with high hematite content, and its effective utilization is difficult due to the special physicochemical properties. In this work, Fe_2_O_3_ in BRM was reduced to Fe_3_O_4_ by biomass, and iron concentrate and high activity tailings were obtained after magnetic separation. The pozzolanic activity and hydration characteristics of the tailings were systematically studied. The results showed that the relatively stable polymerization structures of Si-O and Al-O in BRM are destroyed under the effect of biomass reduction at 650 °C, and some fracture bonds and activation points are formed in the structures. The aluminosilicate phases in the BRM were easy to transform into the active substances of Si and Al. The pozzolanic activity of tailings is greatly improved, and its pozzolanic activity index is 91%. High polymerization degree of gel and ettringite are formed since more active substances and alkali in the tailings promote the hydration reaction of cement-based cementitious materials, which made cementitious materials have dense matrix, good mechanical properties, and environmental performance. This work has realized the full quantitative utilization of BRM and provided a feasible way for the resource utilization of BRM.

## 1. Introduction

BRM is a kind of strong alkaline industrial solid waste, which is discharged from the process of alumina production from bauxite. Normally, 1.0–1.5 tons of BRM will be produced for every ton of alumina [1,2,3]. The emission of BRM is increasing rapidly with the increase of global alumina production. In 2018, about 160 million tons of BRM were produced globally, and the current global storage capacity of BRM is about 4 billion tons [4,5,6]. It is worth noting that a large amount of BRM is currently stacked in the BRM dam since the comprehensive utilization rate of BRM is only about 5% [4]. BRM has a high specific surface area, and fine BRM particles will float with the wind after weathering, causing haze, which seriously affect the normal breathing activities of human and animals. Furthermore, if the BRM storage yard is destroyed, the BRM leachate with high alkalinity will cause soil salinization, disturb the normal growth of plant roots, and seriously damage the ecological environment. Therefore, it is urgent to research methods to improve the utilization rate of BRM.

One of the ways to significantly improve the comprehensive utilization rate of BRM is to use BRM as a supplementary cementitious material to prepare building materials [1,7,8,9]. The current research results showed that most of the phases in red mud have little reactivity in the mixture with cement [5], and the utilization rate of BRM as a supplementary cementitious material is about 10% due to its low pozzolanic activity and high sodium alkali content [4]. There are two main reasons for the low pozzolanic activity of BRM: 1. The content of silicon and aluminum in BRM is low; 2. most of the silicon and aluminum in BRM is inert. Therefore, the pozzolanic activity of BRM can be effectively improved by increasing the content of silicon and aluminum and activating it. One of the effective ways to activate BRM to improve its pozzolanic activity is to calcine it. The purpose of calcination is to decompose some inert phases (such as gibbsite and Katoite, etc.) in red mud and convert them into phases that are easy to react with water or OH^−^ [7,8,9]. The active silica alumina minerals produced by calcined red mud will be dissolved in water or OH^−^ and participate in the reaction of cement to form C-A-S-H gel [4]. Therefore, the general consensus seems to be that the prerequisite of red mud reactivity is calcination, otherwise the inert substances in red mud will not participate in the hydration reaction of cement, which leads to its low utilization in cement. In addition, the content of silicon and aluminum can be effectively increased by extracting the iron oxide in BRM as BRM contains more iron oxide (about 40%). In recent years, although the global iron and steel industry has developed rapidly, iron ore resources have become less and less, and iron ore grades have become lower and lower. In order to alleviate this problem, many researchers have studied the iron recovery in BRM. Zhu et al. [10] blended BRM and Na_2_CO_3_, then added soft coal as a reducing agent after drying, roasted at 1050 °C for 80 min, and then ground the roasted product and magnetically separated the iron from it. Liu et al. [11] used low ash coal char with a carbon content of 92.9% as a reducing agent. After the BRM was roasted under inert gas, the Na and Al in the BRM were recovered by water leaching, and the residue was finally recovered iron by magnetic separation. Borra et al. [12] used graphite powder as a reducing agent, wollastonite powder as a fluxing additive, and recovered iron in BRM through a reduction smelting process in an argon atmosphere. Samouhos et al. [13] used hydrogen as a reducing agent to convert hematite into magnetite at 480 °C, and extract magnetite by wet magnetic separation. The above researches on extracting iron from BRM have achieved good results. However, there are few investigates on tailings after iron extracting from BRM.

Using biomass to reduce BRM can effectively achieve the goal of energy conservation and clean production as biomass is a renewable resource with less pollution compared with traditional reducing agents such as coal and carbon. Our research group previously studied the reduction of BRM by biomass, which can reduce all iron oxide to magnetite, and then separate magnetite from BRM by magnetic separation [4]. An important phenomenon was discovered that 650 °C is not only the optimal reduction temperature, but also the temperature at which the thermal calcination method maximizes the pozzolanic activity of BRM. In our previous report [4], the magnetization mechanism of Fe_2_O_3_ in BRM to magnetite by biomass was analyzed in depth. However, the increasing mechanism of pozzolanic activity of BRM in the process of reducing the iron component of BRM by biomass has not been studied in depth. Therefore, elucidating the increasing mechanism of pozzolanic activity can give a method for BRM research as a supplementary cementitious material.

The pozzolanic activity and hydration characteristics of tailings after iron extraction from biomass magnetized BRM have been deeply investigated. The influence of tailings as supplementary cementitious materials on the properties and microstructure of cementitious materials was explored. The microstructure of the cementitious materials was characterized and the mechanism of enhancing the pozzolanic activity of tailings is revealed.

## 2. Materials and Methods

BRM was obtained from an aluminum plant in Shanxi, China, and the production method was the Bayer process. The BRM was dried, ground, and sieved to obtain a particle size range of 0–0.074 mm. Biomass (bamboo powder) was obtained from a company in Beijing, China, which was broken to a particle size range of 0–2 mm. The cement (42.5) was selected from a cement company in Hebei, China. The XRF was used to analyze the main chemical constituents of BRM and cement, as shown in Table 1. The main chemical constituents of BRM are Fe_2_O_3_, Al_2_O_3_, SiO_2_, Na_2_O, and a small amount of CaO, whereas the content of Fe_2_O_3_ is 26.57%.

The selected bamboo powder is air-dried, crushed, and sieved according to the particle size. The elemental analysis of bamboo powder was analyzed according to the standard GB/T212-2008 [14], and Table 2 shows the results. The volatile matter in the bamboo powder accounts for the largest proportion and the lowest ash content.

According to our previous research [4], the BRM and bamboo powder were uniformly mixed according to the set ratio (5:1), and a cylindrical test piece of φ 8 × 8 mm was prepared under the pressure of 20 MPa, and then the test pieces were placed in the center of the tubular furnace. A high-purity N_2_ gas (flow rate: 100 mL·min^−1^) was introduced into the tubular furnace, and then the tubular furnace was heated at 10 °C/min to 650 °C and kept for 30 min, cooled to room temperature under a nitrogen atmosphere. The Fe_3_O_4_ in the BRM is extracted by the method of magnetic separation to obtain Tailings. The chemical composition of Tailings is shown in Table S1 (see Supplementary Materials). The particle size distribution of Tailings is shown in Figure S1 (see Supplementary Materials). In addition, BRM was calcined at 650 °C in the tubular furnace to obtain calcined BRM at 650 °C (CBRM) as a control group.

**Figure 1:**
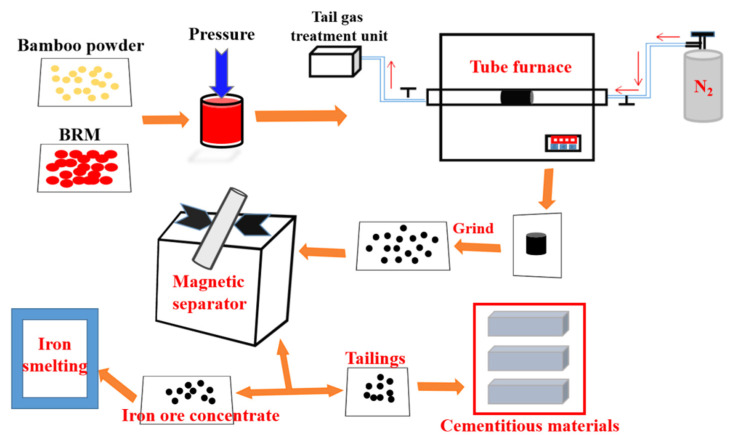
The experimental processes flow.

BRM, CBRM, and Tailings are used to prepare cementitious materials according to the proportion in Table 3. Referring to GB/T 17671-1999 [15], cement mortar experiments were carried out with 40 × 40 × 160 mm samples, the water-cement ratio: w/c = 0.5. Demoulding after curing for a day at (20 ± 1) °C, R.H. = (95 ± 1)% and then curing for the corresponding age at the same condition. The preparation process was shown in Figure 1. In addition, in order to study the hydration behavior of cementitious materials, the pastes of BRM-cement, CBRM-cement, and Tailings-cement were prepared.

The X-ray diffraction (XRD) of the sample was tested with the X-ray diffractometer (D8 ADVANCE, Karlsruhe, Germany) of the German Bruker company. The thermogravimetry (TG) was tested by NETZSCH STA 449F5 (Serb, Germany). The structure and chemical bonds of the sample were characterized by Fourier transform infrared spectrometer (FTIR, Nicolet IS 10, Karlsruhe, Germany). The scanning electron microscope-energy dispersive spectrometer (SEM-EDS, SU8020) was tested to observe and analyze the micromorphology of the sample. The ^29^Si nuclear magnetic resonance (NMR) was tested with Bruker AVANCE III instruments of the German Bruker company (Karlsruhe, Germany). The pore structure of the sample was analyzed by mercury intrusion porosimetry (MIP, AutoPore IV 9500, Atlanta, GA, USA).

The pozzolanic activity index is calculated in accordance with the Chinese standard (GB/T12957-2005) “Test method for activity of industrial waste slag used as addition to cement” [16]. Thirty percent of BRM was added into the cement and prepared the red mud-cement mortar, and the compressive strength at 28 d of red mud-cement mortar was tested. The pozzolanic activity index was calculated by the following formula:

Pozzolanic activity index = R_1_/R_2_, (1)

where R_1_ is the compressive strength at 28 d of red mud-cement mortar and R_2_ is the compressive strength at 28 d of cement mortar.

The pozzolanic activity of BRM is positively related to its the content of amorphous phase. The amorphous phase structure of BRM is composed of Si and Al tetrahedrons, which are connected by bridging oxygen bonds in two ways: Si-O-Si and Si-O-Al. Si (Al) can be divided into structures of SiQ^0^, SiQ^1^, SiQ^2^, SiQ^3^, and SiQ^4^ according to the number of bridging oxygen bonds. The aluminosilicate frameworks in BRM have different degrees of polymerization. The degree of polymerization reflects the crystallization trend of aluminosilicate. Zhang [17] proposed the concept of relative bridging oxygen number (RBO) to evaluate the degree of polymerization, and proposed the calculation formula of the relative bridging oxygen number.

(2) $$\mathrm{RBO}=\frac{1}{4}(1\;\times \;\frac{Q^{1}}{\sum Q^{n}}+2\;\times \;\frac{Q^{2}}{\sum Q^{n}}+3\;\times \;\frac{Q^{3}}{\sum Q^{n}}+4\;\times \;\frac{Q^{4}}{\sum Q^{n}})=\frac{1}{4}\frac{\sum n\cdot Q^{n}}{\sum Q^{n}},$$

where Q^n^ is the relative area of the corresponding formant.

The toxicity leaching tests of cementitious materials were determined by the European Standard EN12457. The leaching tests were carried on by mixing 100 g of cementitious materials with 1 L deionized water in a 1 L container made of polypropylene and subjected to a mixing system. Mixing took place for 24 ± 0.5 h at 30 rpm, and then kept in balance. Vacuum filtration was next. Lastly, the filtrate was acidified (pH ≈ 2.5–3) and the ion concentrations of Na, Cr, Cd, Pb, and As in the filtrate were measured by the inductive coupled plasma emission spectrometer (ICP-MS).

## 3. Results and Discussion

### 3.1. Mechanical Properties and Pozzolanic Activity

**Figure 2:**
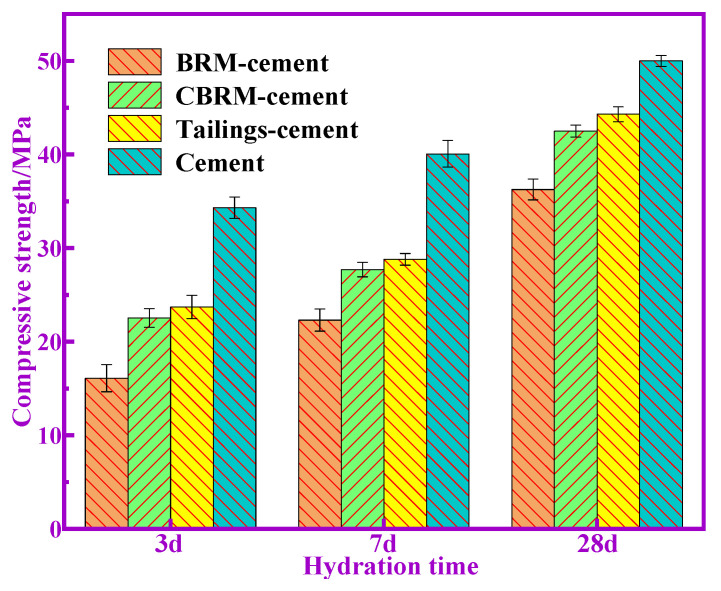
Compressive strength of cementitious materials.

**Figure 3:**
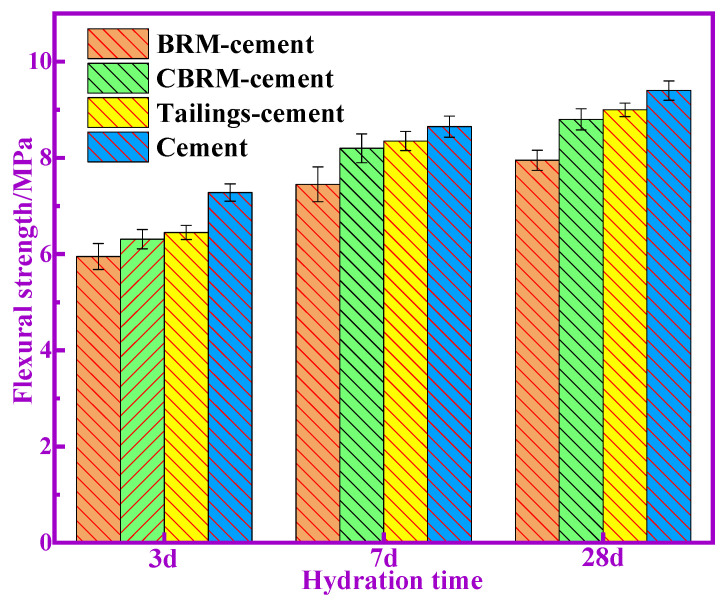
Flexural strength of cementitious materials.

The compressive and flexural strength of BRM-cement, CBRM-cement, and Tailings-cement at 3, 7, and 28 d are shown in Figure 2 and Figure 3. Here, it can be seen that the compressive and flexural strength of all the samples increase with the increase of hydration time. The compressive strength of Tailings-cement is slightly higher than that of BRM-cement and CBRM-cement. The compressive strength of Tailings-cement at 3, 7, and 28 d is 23.7, 28.8, and 44.3 MPa, respectively, which meet the requirements of level 42.5 of the ordinary Portland cement. In addition, the change trend of flexural strength of all the samples is the same as that of compressive strength. The flexural strength of Tailings-cement at 3, 7, and 28 d is 6.44, 8.37, and 9.01 MPa, respectively, which meet the requirements of level 42.5 of the ordinary Portland cement. It is worth noting that the different compressive strength values of BRM-cement, CBRM-cement, and Tailings-cement indicates that they have different pozzolanic activities.

**Figure 4:**
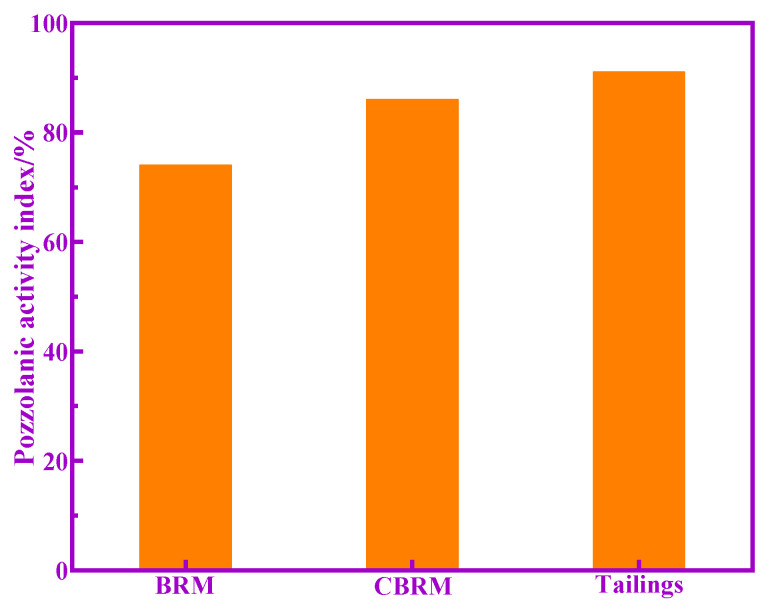
Pozzolanic activity index of BRM, CBRM, and Tailings.

According to the GB/T12957-2005 “Test method for activity of industrial waste slag used as addition to cement”, the pozzolanic activity index of BRM, CBRM, and Tailings is calculated, as shown in Figure 4. Here, it can be seen that the pozzolanic activity index of BRM, CBRM, and Tailings is 74%, 86%, and 91%, respectively. Next, the XRD, TG-DTG, and FTIR of BRM, CBRM, and Tailings were analyzed to reveal the mechanism of enhancing the pozzolanic activity of Tailings compared with BRM and CBRM.

### 3.2. XRD, TG, and FTIR Analysis of BRM, CBRM, and Tailings

**Figure 5:**
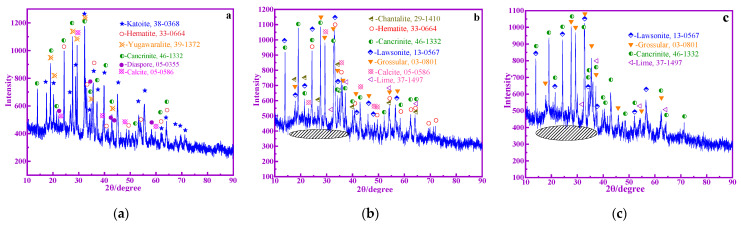
XRD patterns of BRM (**a**), CBRM (**b**), and Tailings (**c**).

The phase composition has an important influence on the pozzolanic activity of BRM. The amorphous phase is the most active phase in BRM. Most of the crystalline components of BRM are considered to be inert and hardly participate in the hydration reaction. The XRD patterns of BRM are shown in Figure 5a. The main mineral compositions of BRM are Ca_3_Al_2_(SiO_4_)(OH)_8_ (Katoite), Fe_2_O_3_ (Hematite), Ca(Si_6_Al_2_)O_16_·4H_2_O (Yugawaralite), CaCO_3_ (Calcite), AlO(OH) (Diaspore), and Na_6_Ca_2_Al_6_Si_6_O_24_(CO_3_)_2_·2H_2_O (Cancrinite). Figure 5b shows the XRD patterns of CBRM, where the main mineral compositions of CBRM are Fe_2_O_3_ (Hematite), CaAl_2_SiO_4_(OH)_4_ (Chantalite), CaCO_3_ (Calcite), CaAl_2_Si_2_O_7_(OH)_2_·H_2_O (Lawsonite), CaO (Lime), and Na_6_Ca_2_Al_6_Si_6_O_24_(CO_3_)_2_·2H_2_O (Cancrinite). The results show that Chantalite, lime, and lawsonite are the newly formed phase, and the phase of Fe_2_O_3_ remains unchanged. Compared with Figure 5a, the diffraction peak intensity of CBRM crystals decrease gradually, especially for cancrinite and calcite. The decrease of the peak intensity of the crystals reveal the appearance of amorphous substances, and the amorphous peaks of 15° to 40° become larger. It shows that some aluminosilicate crystals are decomposed and transformed into active substances of silicon and aluminum, which may be the reason why the pozzolanic activity of CBRM is higher than that of BRM. Figure 5c shows the XRD patterns of Tailings, where the main mineral compositions of Tailings are CaAl_2_Si_2_O_7_(OH)_2_·H_2_O (Lawsonite), Na_6_Ca_2_Al_6_Si_6_O_24_(CO_3_)_2_·2H_2_O (Cancrinite), CaO (Lime) and Ca_3_Al_2_(Si_3_O_4_, CO_3_, OH)_3_ (Grossular). Compared with CBRM, Fe_2_O_3_ is almost completely transformed into Fe_3_O_4_ and separated by magnetic separation, and the diffraction peak intensity of all the crystals in Tailings decrease gradually. The amorphous peaks at 15° to 40° become significantly larger, which indicate that some aluminosilicate phases are transformed into active substances of silicon and aluminum under the action of biomass. This may be the reason why the pozzolanic activity of Tailings is higher than that of CBRM. The results showed that the amorphization degree of each crystal phase in BRM, CBRM, and Tailings is different, and its contribution to the pozzolanic activity is also different. The existence of bamboo powder is conducive to the improvement of amorphization degree of all crystal phases in BRM.

**Figure 6:**
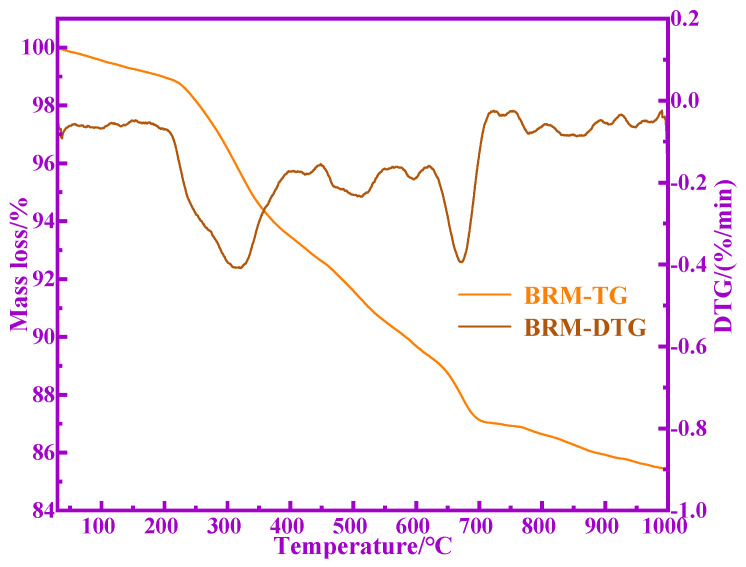
TG-DTG curves of BRM.

The TG-DTG curves of BRM are shown in Figure 6. Here, it can be seen that the continuous mass loss of BRM occurs at 0–1000 °C and the mass loss rate is 14.63%. The mass loss rate in the range of 0–225 °C and 700–1000 °C is slower than that in the range of 225–700 °C. The DTG curve shows that BRM mainly contains two obvious mass loss stages in the heating process. The first stage is 30–500 °C, which is mainly the removal of physically adsorbed water and chemically bound water in BRM. A large amount of physically adsorbed water has been removed during the drying process as the BRM was dried at 100 °C before the experiment, only 0.26% of the physically adsorbed water is removed within the range of 30 °C to 105 °C. The weight loss rate is 7.92% in the range of 105–500 °C, which is mainly the removal of chemically bound water. The second stage is located in the temperature range of 500 to 770 °C, and the weight loss rate is 4.86%, which is mainly caused by the decomposition of aluminosilicate-carbonate in the BRM to release CO_2_.

**Figure 7:**
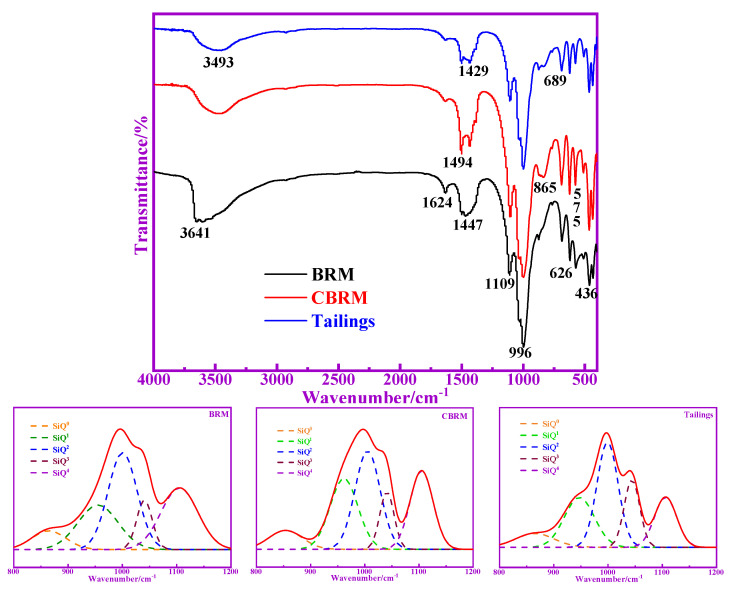
FTIR spectra of BRM, CBRM, and Tailings.

The FTIR spectra of BRM, CBRM, and Tailings are shown in Figure 7. The absorption peak of 3641 cm^−1^ corresponds to the telescopic vibration of free -OH. The absorption peak of 3493 cm^−1^ corresponds to the telescopic vibration of associated -OH. After calcination at 650 °C, the free -OH in the BRM is converted to an associated -OH, which indicates a certain amount of hydrogen bond. The absorption peak of 1624 cm^−1^ corresponds to the telescopic vibration of C-O, and about 1450 cm^−1^ corresponds to the antisymmetric telescopic vibration of C-O. The absorption peak in the range of 800–1200 cm^−1^ is asymmetric tensile vibration of Si-O-Si or Si-O-Al connected with tetrahedron of [SiO_4_] or [AlO_4_]^−^ [18]. The peak at 1090 cm^−1^ is caused by the tensile vibration of O-Si-(Si). The peak at 996 cm^−1^ is caused by the tensile vibration of Si-O (Al). The peak at 865 cm^−1^ is caused by the tensile vibration of Si-O-. The absorption peak in the range of 800–600 cm^−1^ is symmetrical to telescopic vibrations between Si-O-(Si, Al) in tetrahedron of [SiO_4_] or [AlO_4_]^−^. The absorption peak of 575 cm^−1^ may be related to the rings of silicon oxygen and aluminum oxygen. The peak in the range of 500–400 cm^−1^ is caused by the bending vibration of Si-O-Si or Si-O-Al. It is worth noting that the FTIR spectra at 800–1200 cm^−1^ of BRM, CBRM, and Tailings are similar, but the area of peak has some differences. Based on the existing research results, the characteristic peaks of SiQ^0^, SiQ^1^, SiQ^2^, SiQ^3^, and SiQ^4^ are 840–900 cm^−1^, 900–950 cm^−1^, 950–1030 cm^−1^, 1030–1100 cm^−1^, and 1100–1200 cm^−1^, respectively. The Origin software was used to separate and fit the peaks of 800–1200 cm^−1^, whereas the peaks area and RBO were calculated. The related peaks information are shown in Figure 7 and Table 4. It can be seen from Table 4 that the content of SiQ^4^ in BRM is higher than that in CBRM and Tailings. Combined with the XRD results, SiQ^4^ in BRM represents the structure with more Si-O-Si (Al), which indicates that its crystal has a high degree of polymerization and stable chemical properties. The SiQ^4^ of CBRM is lower than that of BRM, which indicates that some stable SiQ^4^ structures in BRM are depolymerized and low degrees of polymerization substances are formed at 650 °C. It is worth noting that SiQ^4^ of Tailings is significantly lower than that of CBRM, which indicates that bamboo powder promotes the depolymerization of SiQ^4^ structure at 650 °C. The alkali system of cement hydration reaction is conducive to the dissolution of silicon and aluminum components in Tailings, which makes the pozzolanic activity of Tailings higher than that of BRM and CBRM. The results showed that the Si-O-Si (Al) bond of aluminosilicate in BRM is destroyed after being reduced by biomass, and the polymerization degree of aluminosilicate decreases, which leads to the increase of the active substance of silicon and aluminum in Tailings.

In summary, the diaspore in BRM will decompose into Al_2_O_3_ at about 500 °C, there is no corresponding characteristic peak in XRD of CBRM and Tailings, which indicates that alumina may be amorphous. In the range of 300–600 °C, Katoite and yugawaralite begin to remove part of the -OH and gradually transform into Chantalite and lawsonite. The free -OH of aluminosilicates in BRM is removed, which is conducive to the depolymerization of aluminosilicates and the reduction of polymerization degree of CRBM. Cancrinite in BRM begins to decompose into lime and amorphous aluminosilicate at about 650 °C. More importantly, the reduction gases of lime and H_2_ produced by pyrolysis of bamboo powder can transform all hematite in BRM into magnetite, and also promote the decomposition of aluminosilicate phase (cancrinite), which produces CaO and active silicon and aluminum. At the same time, the FTIR spectra of Tailings show that the Si-O bond and Al-O bond are broken, which indicate that the polymerization degree of aluminosilicate is reduced, and the pozzolanic activity of Tailings is improved.

### 3.3. Hydration Characteristics of BRM, CBRM, and Tailings

#### 3.3.1. XRD Analysis of Cement-Based Cementitious Materials

**Figure 8:**
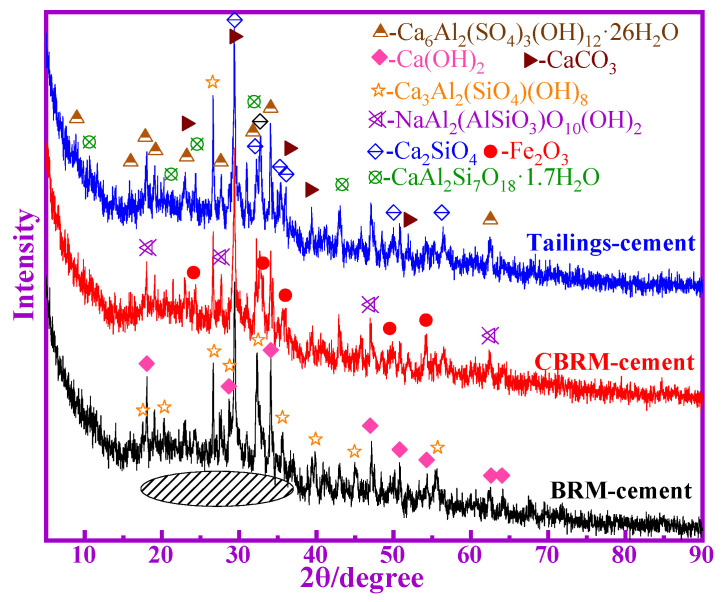
The XRD patterns of BRM-cement, CBRM-cement, and Tailings-cement.

The XRD patterns of hardened pastes of BRM-cement, CBRM-cement, and Tailings-cement at 28 d are shown in Figure 8. Here, it can be seen that the ettringite and aluminosilicate are generated in BRM-cement, CBRM-cement, and Tailings-cement, which promote the strength development. It is worth noting that the peaks of Fe_2_O_3_ appear in the BRM-cement and CBRM-cement, which indicate that Fe_2_O_3_ does not participate in the hydration reaction of cementitious materials. Therefore, the high content of Fe_2_O_3_ in BRM is one of the main reasons for its low pozzolanic activity, which also shows that one of the reasons for the increase in pozzolanic activity of Tailings is that the Fe_2_O_3_ transition into Fe_3_O_4_ is magnetically separated. Compared with the XRD of BRM, CBRM, and Tailings, the center of miscellaneous diffusion peaks (15–40°) of cementitious materials shift to the right, which indicates the formation of amorphous phase in the product (C-S-H gel and C-A-S-H gel) [18]. The peaks with Ca(OH)_2_ appear in the XRD patterns of BRM-cement, CBRM-cement, and Tailings-cement. However, the intensity of the peak is different due to the different extent of hydration reaction. Ca(OH)_2_ is formed by the reaction of CaO with H_2_O, which can generate a pozzolanic reaction with the active SiO_2_ and Al_2_O_3_ in cementitious materials, and lead to the formation of gel and ettringite [19]. Therefore, compared to BRM-cement, the CBRM-cement and Tailings-cement get better activation and produce more active SiO_2_ and Al_2_O_3_ and with stronger pozzolanic reactivity.

#### 3.3.2. FTIR Analysis of Cement-Based Cementitious Materials

**Figure 9:**
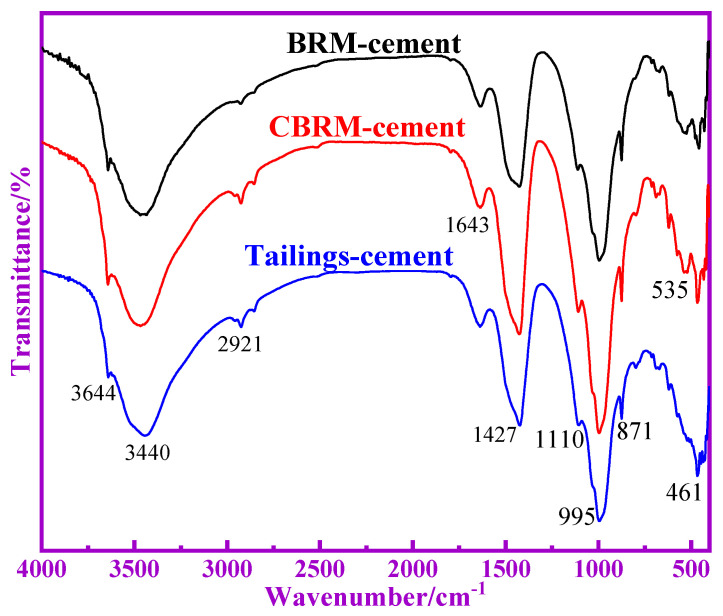
FTIR absorption spectra of BRM-cement, CBRM-cement, and Tailings-cement.

Figure 9 shows the FTIR absorption spectra of BRM-cement, CBRM-cement, and Tailings-cement at 28 d. The absorption peak in the range of 800–1200 cm^−1^ is asymmetric tensile vibration of Si-O-Si or Si-O-Al connected with tetrahedron of [SiO_4_] or [AlO_4_]^−^. The bending vibration at 3440 cm^−1^ is an Al-OH chemical bond, which represents [Al(OH)_6_]^3−^ structures of the octahedra of ettringite [19], and the absorption spectrum of Tailings-cement is the widest. The weak absorption peaks at 1643 cm^−1^ indicate the vibration spectrum of H-O-H. The vibration peak observed from 871 cm^−1^ is caused by the asymmetric stretching vibration of Si-OH and 461 cm^−1^ represents an absorption spectra for Si-O [20].

The absorption peaks at 3643 cm^−1^ and 1637 cm^−1^ indicate the telescopic and free-water bending vibrations of Ca(OH)_2_, respectively [21]. The absorption peaks of Tailings-cement at 3644 cm^−1^ and 1643 cm^−1^ weaken, implying that more Ca(OH)_2_ is involved in the hydration reaction, and more free water is changed to bound water during the reaction. Compared with the BRM-cement and CBRM-cement, the absorption peak value of spectra at 3440 cm^−1^ has an increasing trend relatively, implying that a much higher amount of [Al(OH)_6_]^3−^ is generated in addition to the hydration products. This is similar to the results of XRD analysis. The hydration degree of Tailings-cement is high, and more hydration products are formed.

#### 3.3.3. Pore Structure Analysis of Cement-Based Cementitious Materials

Characterization of the pore is an important component of the microstructure of cementitious materials. The pore diameter distribution, the number of gel pores, and the gel density of the cementitious materials affect the strength of the cementitious materials. The MIP results are shown in Table 5. It is generally believed that in cementitious materials, the pores that are larger than 1 um are harmful, and cementitious materials with fewer harmful pores have higher mechanical properties [18,19,20,21,22]. It is obvious that the Tailings-cement owns fewer harmful pores, and the CBRM-cement takes the second place. As is well known, the pore sizes ≤10 nm are called gel pores and the gel pores were generated with C-S-H gel and C(N)-A-S-H gel for BRM-cement, CBRM-cement, and Tailings-cement [23]. The pozzolanic activity of BRM, CBRM, and Tailings can be inferred from the content of gel pores. The gel pores in BRM-cement, CBRM-cement, and Tailings-cement are 32.82%, 34.32%, and 35.94%, respectively. The amount of gel pores is highest in Tailings-cement, which indicates that the gel produced in Tailings-cement is the most and the Tailings-cement has higher pozzolanic reactivity. The total pore volume of cementitious material is small, which indicates that its gel density is large and its mechanical properties are high [24]. The total pore volume, average pore diameter, and porosity of Tailings-cement are best, which indicate that the Tailings-cement produces more hydration products.

**Figure 10:**
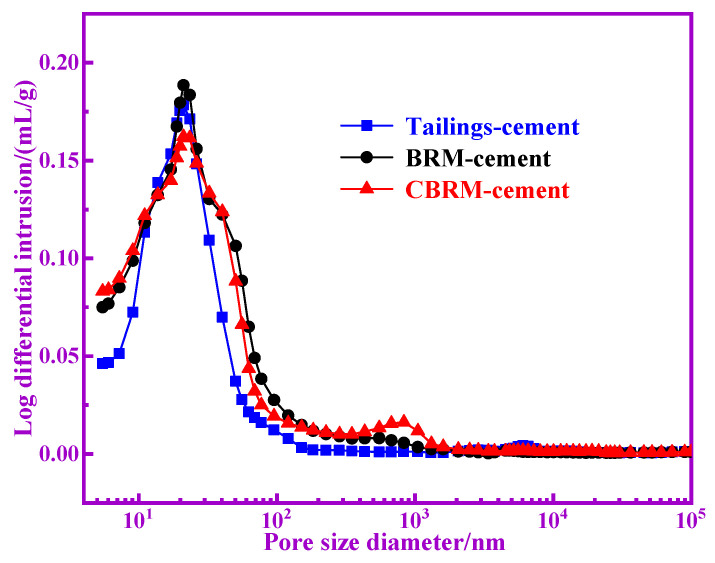
The curves of log differential invasion of BRM-cement, CBRM-cement, and Tailings-cement.

**Figure 11:**
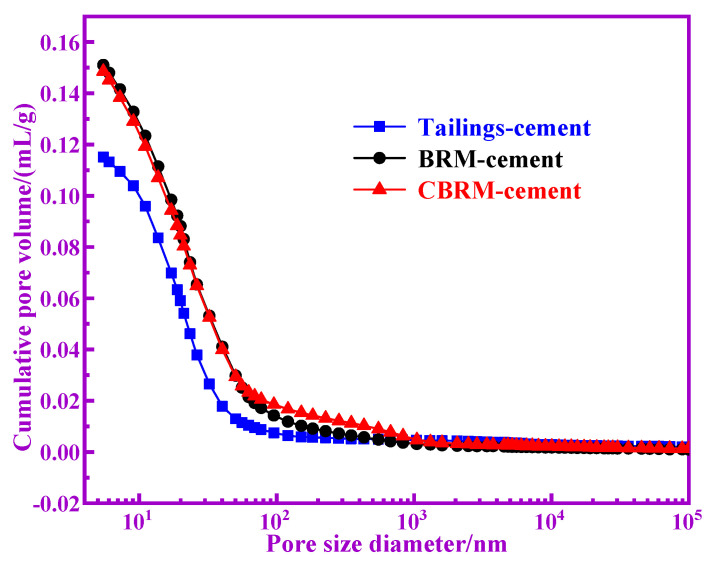
The curves of cumulative pore volume of BRM-cement, CBRM-cement, and Tailings-cement.

The curves of log differential invasion and cumulative pore volume of BRM-cement, CBRM-cement, and Tailings-cement are shown in Figure 10 and Figure 11. The pore size distribution curves of BRM-cement, CBRM-cement, and Tailings-cement are approximately at normal distribution and the cumulative pore volume of Tailings-cement is the smallest. It indicates that the best pore and dense structure was obtained by the Tailings-cement and the Tailings-cement has the best strength.

#### 3.3.4. Thermal Analysis of Cement-Based Cementitious Materials

**Figure 12:**
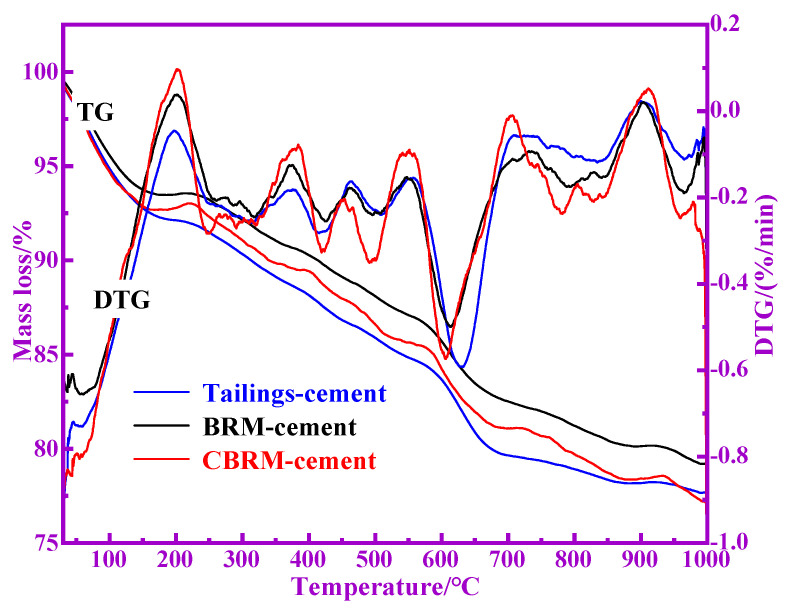
The TG-DTG curves of BRM-cement, CBRM-cement, and Tailings-cement.

The TG-DTG curves of the hardened paste of BRM-cement, CBRM-cement, and Tailings-cement at 28 d are shown in Figure 12. The mass loss of BRM-cement, CBRM-cement, and Tailings-cement is continuous with the increase of temperature, and their total mass loss is 20.86%, 23.02%, and 22.40%, respectively. The mass loss of BRM-cement, CBRM-cement, and Tailings-cement can be divided into four main ranges: The first range is 30–100 °C, which represents the mass loss of free water in hardened paste. The second range is 100–300 °C, which represents the mass loss of bound water of C-S-H gel, C(N)-A-S-H gel, and ettringite in BRM-cement, CBRM-cement, and Tailings-cement [25]. The third temperature range is about 300–750 °C, which represents the decomposition of Si(Al)-OH structure and Ca(OH)_2_ in BRM-cement, CBRM-cement, and Tailings-cement [26]. The fourth temperature range is about 750–1000 °C, which corresponds to the decomposition of calcium carbonate in BRM-cement, CBRM-cement, and Tailings-cement.

The mass loss parameters of BRM-cement, CBRM-cement, and Tailings-cement in different ranges are shown in Table 6. The mass loss of BRM-cement, CBRM-cement, and Tailings-cement in different ranges can indirectly reflect the corresponding content of hydration products. The mass loss of BRM-cement, CBRM-cement, and Tailings-cement in the range of 30–100 °C is 4.44%, 5.32%, and 5.14%, respectively. The mass loss of BRM-cement, CBRM-cement, and Tailings-cement in the range of 100–300 °C is 3.40%, 3.61%, and 4.54%, respectively. It indicates that the total amount of C-S-H gel, C(N)-A-S-H gel, and ettringite in the hydration products of Tailings-cement is the largest. The mass loss of BRM-cement, CBRM-cement, and Tailings-cement in the range of 300–750 °C is 10.19%, 10.40%, and 10.96%, respectively, which indicates that the total amount of Si(Al)-OH structure and Ca(OH)_2_ of Tailings-cement are the most. It is worth noting that the XRD patterns show that the content of Ca(OH)_2_ in Tailings-cement is less, which indicates that Tailings-cement has more Si(Al)-OH structure. Therefore, the active substance of silicon and aluminum in the Tailings-cement participate in the hydration reaction. The mass loss of BRM-cement, CBRM-cement, and Tailings-cement in the range of 750–1000 °C is 2.83%, 3.69%, and 1.76%, respectively. Compared with BRM-cement and CBRM-cement, the carbonization degree of Tailings-cement is smaller, which indicates that the hydration reaction of Tailings-cement is more thorough.

#### 3.3.5. The ^29^Si MAS NMR Analysis of Cement-Based Cementitious Materials

The tool to characterize amorphous and weak amorphous phases of Si in cementitious materials were characterized by ^29^Si NMR. In this research, the ^29^Si NMR spectra were used to study a deeper structural transformation of cementitious materials [27]. It is worth noting that the polymerization degree or depolymerization can be shown by the change of the number of coordination bridge oxygen bonds during the hydration reaction. The relative bridge oxygen (RBO) number sums up to an accurate calculation the degree of the polymerization [SiO_4_] [28].

**Figure 13:**
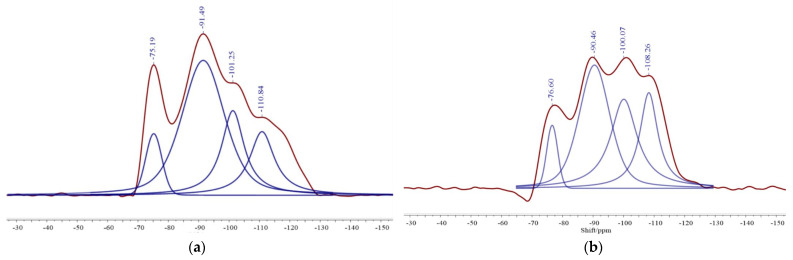
The ^29^Si NMR spectra of CBRM-cement (**a**) and Tailings-cement (**b**).

The chemical shift range of the structural unit of Q^n^ in CBRM-cement and Tailings-cement are shown in Figure 13 and Table 7. There are four resonance peaks in the hydration products of CBRM-cement and Tailings-cement, which represent the bridging oxygen structure with different Si-O. Al replaces Si results in a chemical shift to 3 to 5 ppm, the values significantly more positive [29]. As a result, Q^3^(1Al) is characterized by peaks approaching 91 ppm, while the chemical shift around 100 ppm is Q^4^(1Al). It can be seen that the chemical shifts in CBRM-cement are −75.91, −91.49, −101.25, and −110.84 ppm, respectively. The chemical shift of Tailings-cement is similar to CBRM-cement. The main peaks are Q^0^, Q^3^(1Al), Q^4^(1Al), and Q^4^, respectively. Q^0^ is mainly caused by C_2_S or C_3_S in raw materials, indicating that some Si does not participate in the hydration reaction. Q^3^(1Al), Q^4^(1Al), and Q^4^ are caused by C-S-H gel, C-A-S-H gel, and aluminosilicate [27]. According to the observation, the peak intensity of Q^0^ in CBRM-cement is significantly stronger than that in Tailings-cement, which means that the hydration degree of Tailings-cement is more complete. The relative area of CBRM-cement and Tailings-cement is shown in Table 8, it is observed here that the relative area of Q^4^(1Al) and Q^4^ in Tailings-cement are greater than the CBRM-cement, which proves that more gel products generated in Tailings-cement, and then showed better activation [30]. Meanwhile, the RBO of CBRM-cement and Tailings-cement are 60.07% and 62.5%, respectively. It states that the polymeric level of the [SiO_4_] tetrahedra in Tailings-cement is better than CBRM-cement, and the Tailings-cement has a better polymerization structure. It is worth noting that [AlO_4_]^−^ in the C-A-S-H gel has a charge effect on cations, while the [AlO_4_]^−^ structure content in the Tailings-cement is more, which indicates that Na^+^ in the red mud can be effectively solidified.

#### 3.3.6. SEM-EDS Analysis of Cement-Based Cementitious Materials

**Figure 14:**
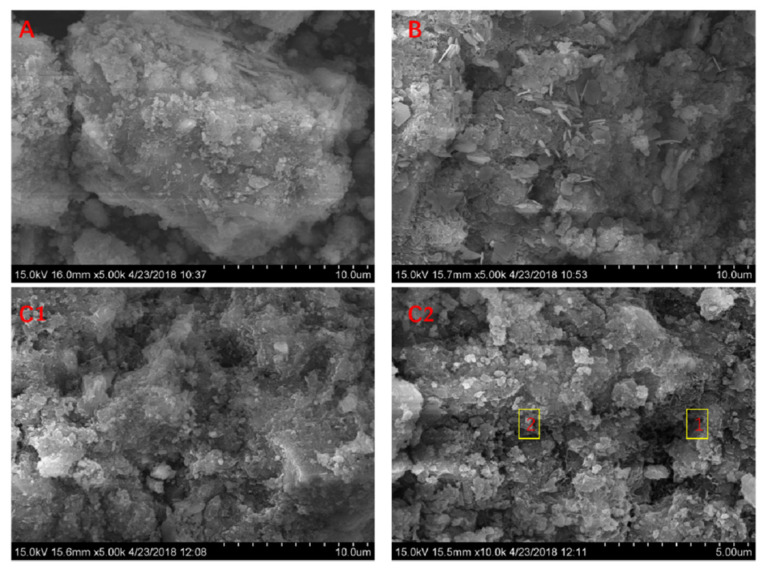
SEM pictures of BRM-cement (**A**), CBRM-cement (**B**), Tailings-cement (**C1**) and EDS of Tailings-cement (**C2**).

SEM can observe the morphology of cementitious materials prepared at different hydration ages and with BRM, CBRM or Tailings as raw materials. Meanwhile, chemical elements of the characteristic points were measured and analyzed by EDS. Combined with the energy spectrum analysis, the composition of the surface materials of the sample can be identified, and the factors influencing the strength growth of the net slurry sample can be researched. Figure 14 shows the SEM pictures and EDS of BRM-cement, CBRM-cement, and Tailings-cement at 28 d.

Table 9 shows the elements distribution of regions 1 and 2 in Figure 14(C2). It can be seen from Figure 14 and Table 9 that there are fibrous C-A-S-H gel and rod ettringite in CBRM-cement and Tailings-cement. Only a few rod ettringites can be observed in BRM-cement. It can be clearly observed that ettringite is mostly distributed in the pores and pits, which makes the structure of slurry more compact [20]. There is more fibrous C-A-S-H gel interweaved in the network structure, and ettringite is connected together to reduce porosity, which makes the cementitious materials have better cementitious properties [17]. It is worth noting that there are more hydration products and lower pores of Tailings-cement, which have the highest compressive and flexural strength.

#### 3.3.7. Environmental Performance of Cement-Based Cementitious Materials

Leaching test findings have been contrasted against the EU Directive on drinking water quality (98/83/EC). Table 10 shows that the hazardous metals Na, Cr, and Pb comply with the standard requirements. According to the previous research results [19,20,21,22], C-S-H gel, C-A-S-H gel, and ettringite produced in cementitious materials can effectively solidify Na and heavy metal ions. It can be seen from Table 10 that the heavy metal ions were not detected in the cementitious materials at 28 d, and the leaching concentration of Na in BRM-cement, CBRM-cement, and Tailings-cement is 47.261, 28.543, and 10.397 mg/L, respectively, which indicates that more active silicon and aluminum in Tailings are involved in the hydration reaction of cementitious materials, and the gel and ettringite can effectively solidify Na and heavy metal ions. Therefore, the cementitious materials are environmentally friendly, and the pozzolanic activity and environmental performance of Tailings meet the requirements of supplementary cementitious materials for cement. It can be seen from the above results that the method of using bamboo powder to reduce BRM in order to extract iron to synergistically activate the pozzolanic activity of its Tailings is very promising.

## 4. Conclusions

The pozzolanic activity and hydration characteristics of Tailings after iron extraction in BRM by biomass reduction were systematically studied, and the following conclusions were obtained:

The relatively stable polymerization structures of Si-O and Al-O in BRM were destroyed under the effect of biomass reduction at 650 °C, and some fracture bonds and activation points are formed in the structures. The inert aluminosilicate in BRM was easy to be transformed into active substances, which made the Tailings have high pozzolanic activity. The pozzolanic activity index of the Tailings was 91%, which was higher than that of BRM. As a supplementary cementing material, the Tailings can meet the requirements of ordinary Portland cement 42.5 in terms of compressive and flexural properties at 3, 7, and 28 d.

The active substances of Si and Al formed the gel structure with a high degree of polymerization during the hydration reaction process, and the alkali in the BRM made the hydration reaction of the system more complete. The cementitious materials prepared from Tailings and cement contain a large amount of C-S-H gel, C(N)-A-S-H gel, and ettringite, which not only play a positive role in improving the properties, but also have a good solidification effect on Na and heavy metal ions. The close combination of gel and ettringite effectively fills the pores to obtain a higher density of the matrix.

Therefore, Hematite in BRM is reduced to magnetite by biomass, and the iron concentrate separated by magnetic separation is used for ironmaking. Additionally, the Tailings can be used to prepare cementitious materials, which can realize the full utilization of BRM, and have an economic and environmental value.

## Figures and Tables

**Table 1:** The chemical constituents of BRM and cement (wt%).

Oxides	Fe_2_O_3_	Al_2_O_3_	SiO_2_	Na_2_O	CaO	TiO_2_	SO_3_	MgO	Other
BRM	26.57	22.96	22.71	11.08	2.17	1.72	1.01	0.13	11.65
Cement	3.36	3.57	15.10	0.27	68.74	0.31	3.30	3.31	2.04

**Table 2:** The performance of bamboo powder (wt%).

Biomass	Elemental Analysis				Industrial Analysis			
	Carbon	Hydrogen	Oxygen	Nitrogen	Moisture	Volatile	Fixed Carbon	Ash
Bamboo powder	46.57	4.92	46.14	1.43	7.85	78.16	13.40	1.83

**Table 3:** Design of different cement mortars (g).

Number	BRM	CBRM	Tailings	Cement	Standard Sand
BRM-cement	135	—	—	315	1350
CBRM-cement	—	135	—	315	1350
Tailings-cement	—	—	135	315	1350
Cement	—	—	—	450	1350

**Table 4:** The RBO of BRM, CBRM, and Tailings.

Sample	Relative Content/%					RBO	R^2^
	Q^0^	Q^1^	Q^2^	Q^3^	Q^4^		
BRM	7.13	18.84	33.22	10.29	30.52	0.596	0.995
CBRM	7.36	22.93	30.84	10.40	28.47	0.574	0.995
Tailings	7.40	20.65	32.98	15.72	23.25	0.567	0.995

**Table 5:** Detailed data of pore structure.

Sample	Total Pore Volume (mL/g)	Average Pore Diameter/nm	Porosity/%	Pore Size Distribution/%			
				<10 nm	10–50 nm	50 nm–1 μm	>1 μm
BRM-cement	0.1510	18.47	25.41	32.82	45.09	14.92	7.17
CBRM-cement	0.1486	18.19	24.63	34.32	47.05	11.87	6.76
Tailings-cement	0.1151	17.40	20.71	35.94	49.71	9.30	5.05

**Table 6:** The mass loss of BRM-cement, CBRM-cement, and Tailings-cement (wt%).

T/°C	BRM-Cement	CBRM-Cement	Tailings-Cement
<100	4.44	5.32	5.14
100–300	3.40	3.61	4.54
300–750	10.19	10.40	10.96
750–1000	2.83	3.69	1.76
Total	20.86	23.02	22.40

**Table 7:** Chemical shift range of the structure unit.

Symbol	SiQ^0^	SiQ^1^	SiQ^2^	SiQ^3^	SiQ^4^
Chemical Shift/ppm	−66 to −77	−77 to −82	−85 to −89	−92 to −100	−103 to −115

**Table 8:** The SiQ^n^ and RBO of CBRM-cement and Tailings-cement.

Sample	Chemical Shift/ppm	Assign	Relative Area	RBO Value
CBRM-cement	−75.19	Q^0^	33	60.07%
	−91.49	Q^3^ (1Al)	100	
	−101.25	Q^4^ (1Al)	38	
	−110.84	Q^4^	40	
Tailings-cement	−76.60	Q^0^	30	62.50%
	−90.46	Q^3^ (1Al)	100	
	−100.07	Q^4^ (1Al)	58	
	−108.26	Q^4^	44	

**Table 9:** Percentage of elements in regions 1 and 2.

Location	Element/%					
	O	Na	Al	Si	Ca	Fe
1	37.12	3.15	8.23	10.24	41.22	0.64
2	40.74	1.63	4.86	16.70	34.49	1.58

**Table 10:** The SiQ^n^ and RBO of CBRM-cement and Tailings-cement.

Content/(mg/L)	Na	Cr	Cd	Pb	As
EU Standard	200	0.05	0.005	0.01	0.01
BRM	892.55	0.125	0.002	0.233	0.104
BRM-cement	47.261	Not detected	Not detected	Not detected	Not detected
CBRM-cement	28.543	Not detected	Not detected	Not detected	Not detected
Tailings-cement	10.397	Not detected	Not detected	Not detected	Not detected

## Data Availability

Data sharing is not applicable to this article.

## Supplementary Materials

The following are available online at https://www.mdpi.com/article/10.3390/ma14143955/s1, Figure S1: the particle distribution of Tailings, Table S1: the chemical constituents of Tailings (wt.%).

## References

[1] Li Z., Zhang J., Li S., Gao Y., Liu C., Qi Y. (2020). Effect of different gypsums on the workability and mechanical properties of red mud-slag based grouting materials. J. Clean. Prod..

[2] Hertel T., Pontikes Y. (2020). Geopolymers, inorganic polymers, alkali-activated materials and hybrid binders from bauxite residue (red mud)–Putting things in perspective. J. Clean. Prod..

[3] Li X., Zhang Q., Mao S. (2021). Investigation of the bond strength and microstructure of the interfacial transition zone between cement paste and aggregate modified by Bayer red mud. J. Hazard. Mater..

[4] Wang Y., Li D., Liu X., Zhang W., Li Z., Li Y., Ren Y., Li H. (2021). Mechanism of magnetizing the Bayer red mud and meanwhile improving the cementitious activity of its tailings by using biomass. J. Clean. Prod..

[5] Mukiza E., Zhang L., Liu X., Zhang N. (2019). Utilization of red mud in road base and subgrade materials: A review. Resour. Conserv. Recycl..

[6] Li Y., Min X., Ke Y., Liu D., Tang C. (2019). Preparation of red mud-based geopolymer materials from MSWI fly ash and red mud by mechanical activation. Waste Manag..

[7] Vigneshwaran S., Uthayakumar M., Arumugaprabu V. (2019). Development and sustainability of industrial waste-based red mud hybrid composites. J. Clean. Prod..

[8] Zhou W., Shi X., Lu X., Qi C., Luan B., Liu F. (2020). The mechanical and microstructural properties of refuse mudstone-GGBS-red mud based geopolymer composites made with sand. Constr. Build. Mater..

[9] Singh S., Aswath M.U., Ranganath R.V. (2018). Effect of mechanical activation of red mud on the strength of geopolymer binder. Constr. Build. Mater..

[10] Zhu D.-Q., Chun T.-J., Pan J., He Z. (2012). Recovery of iron from high-iron red mud by reduction roasting with adding sodium salt. J. Iron Steel Res. Int..

[11] Liu W., Sun S., Zhang L., Jahanshahi S., Yang J. (2012). Experimental and simulative study on phase transformation in Bayer red mud soda-lime roasting system and recovery of Al, Na and Fe. Miner. Eng..

[12] Borra C.R., Blanpain B., Pontikes Y., Binnemans K., Gerven T.V. (2016). Smelting of bauxite residue (red mud) in view of iron and selective rare earths recovery. J. Sustain. Metall..

[13] Samouhos M., Taxiarchou M., Pilatos G., Tsakiridis P.E., Devlin E., Pissas M. (2017). Controlled reduction of red mud by H_2_ followed by magnetic separation. Miner. Eng..

[14] Supervision T. (2008). Proximate Analysis of Coal, in Chinese Code GB/T212-2008.

[15] Supervision T. (1999). Method of Testing Cements-Determination of Strength, in Chinese Code GB/T 17671-1999.

[16] Supervision T. (2005). Test Method for Activity of Industrial Waste Slag Used as Addition to Cement, in Chinese Code GB/T12957-2005.

[17] Zhang J.-X., Sun H.-H., Sun Y.-M., Zhang N. (2009). Correlation between 29 Si polymerization and cementitious activity of coal gangue. J. Zhejiang Univ. Sci. A.

[18] Wang Q., Wang D., Zhuang S. (2017). The soundness of steel slag with different free CaO and MgO contents. Constr. Build. Mater..

[19] Peng D., Wang Y., Liu X., Tang B., Zhang N. (2019). Preparation, characterization, and application of an eco-friendly sand-fixing material largely utilizing coal-based solid waste. J. Hazard. Mater..

[20] Wang Y., Gao S., Liu X., Tang B., Mukiza E., Zhang N. (2019). Preparation of non-sintered permeable bricks using electrolytic manganese residue: Environmental and NH3-N recovery benefits. J. Hazard. Mater..

[21] Zhang Y., Liu X., Xu Y., Tang B., Wang Y., Mukiza E. (2019). Preparation and characterization of cement treated road base material utilizing electrolytic manganese residue. J. Clean. Prod..

[22] Zhang Y., Liu X., Xu Y., Tang B., Wang Y., Mukiza E. (2019). Synergic effects of electrolytic manganese residue-red mud-carbide slag on the road base strength and durability properties. Constr. Build. Mater..

[23] Zhang N., Liu X., Sun H., Li L. (2011). Pozzolanic behaviour of compound-activated red mud-coal gangue mixture. Cem. Concr. Res..

[24] Chen X., Zhu G., Zhou M., Wang J., Chen Q. (2018). Effect of organic polymers on the properties of slag-based geopolymers. Constr. Build. Mater..

[25] Wang Y., Liu X., Zhang W., Li Z., Zhang Y., Li Y., Ren Y. (2020). Effects of Si/Al ratio on the efflorescence and properties of fly ash based geopolymer. J. Clean. Prod..

[26] Hanif A., Parthasarathy P., Ma H., Fan T., Li Z. (2017). Properties improvement of fly ash cenosphere modified cement pastes using nano silica. Cem. Concr. Compos..

[27] Liu X., Zhao X., Yin H., Chen J., Zhang N. (2018). Intermediate-calcium based cementitious materials prepared by MSWI fly ash and other solid wastes: Hydration characteristics and heavy metals solidification behavior. J. Hazard. Mater..

[28] Cabrera J., Rojas M.F. (2001). Mechanism of hydration of the metakaolin–lime–water system. Cem. Concr. Res..

[29] Zain A.M., Shaaban M.G., Mahmud H. (2010). Immobilization of petroleum sludge incorporating portland cement and rice husk ash. Int. J. Chem. Eng. Appl..

[30] Andersen M.D., Jakobsen H.J., Skibsted J. (2004). Characterization of white Portland cement hydration and the CSH structure in the presence of sodium aluminate by ^27^Al and ^29^Si MAS NMR spectroscopy. Cem. Concr. Res..

