# Supplementary material for: Tailings after Iron Extraction in Bayer Red Mud by Biomass Reduction: Pozzolanic Activity and Hydration Characteristics
Source: Materials (Basel). 2021 Jul 15;14(14):3955. doi: 10.3390/ma14143955 (PMC8306629; doi:10.3390/ma14143955)
Supplement: Supplementary file 1 [file materials-14-03955-s001.zip › materials-1290000-supplementary.pdf]

materials

Article

Tailings after Iron Extraction in Bayer Red Mud by Biomass Reduction: Pozzolanic Activity and Hydration Characteristics

The chemical constituents of Tailings is shown in Table S1. Tailings was the materials after iron extraction from BRM. The major chemical constituents of Tailings were Al_2O_3 (31.47%), SiO_2 (28.82%) and CaO (24.35%).

Table S1. The chemical constituents of Tailings (wt.%).

Oxides	Fe_2O_3	Al_2O_3	SiO_2	Na_2O	CaO	TiO_2	MgO	Other
Tailings	5.21	31.47	28.82	5.66	24.35	3.27	0.29	0.93

The particle size distribution was measured by the laser particle size distribution instrument (mastersizer-2000). The test results of Tailings was shown in Figure S1, and d_{10} was 0.89 μm, d_{50} was 4.34 μm, and d_{90} was 33.57 μm.

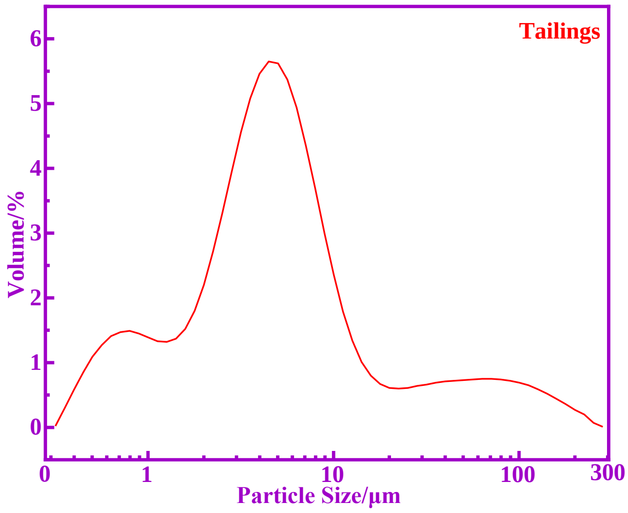

Figure S1. The particle distribution of Tailings.

Materials **2021**, *14*, 3955. https://doi.org/10.3390/ma14143955 www.mdpi.com/journal/materials
